# Efficient leukocyte depletion by a novel microfluidic platform enables the molecular detection and characterization of circulating tumor cells

**DOI:** 10.18632/oncotarget.22549

**Published:** 2017-11-28

**Authors:** Eva Obermayr, Elisabeth Maritschnegg, Christiane Agreiter, Nina Pecha, Paul Speiser, Samir Helmy-Bader, Sabine Danzinger, Michael Krainer, Christian Singer, Robert Zeillinger

**Affiliations:** ^1^ Molecular Oncology Group, Department of Obstetrics and Gynecology, Comprehensive Cancer Center, Medical University of Vienna, Waehringer Guertel 18-20, 1090 Vienna, Austria; ^2^ Department of Obstetrics and Gynecology, Clinical Division for General Gynecology and Gynecological Oncology, Comprehensive Cancer Center, Medical University of Vienna, Waehringer Guertel 18-20, 1090 Vienna, Austria; ^3^ Department of Obstetrics and Gynecology, Division of Senology, Comprehensive Cancer Center, Medical University of Vienna, Waehringer Guertel 18-20, 1090 Vienna, Austria; ^4^ Department of Medicine I, Clinical Division of Oncology, Comprehensive Cancer Center, Medical University of Vienna, Waehringer Guertel 18-20, 1090 Vienna, Austria

**Keywords:** circulating tumor cells, microfluidics, gene expression analysis, ovarian cancer

## Abstract

RT-qPCR is a highly sensitive approach to detect rare transcripts, as derived from circulating tumor cells (CTCs) in the blood of cancer patients. However, the presence of unwanted leukocytes often leads to false positive results. Here, we evaluated whether the micro-fluidic Parsortix™ technology is appropriate to remove these leukocytes and thereby finally to improve the overall approach.

In this study, we established a workflow including the micro-fluidic Parsortix™ technology for the molecular detection of CTC related transcripts. Background levels of *EpCAM*, *PPIC*, *TUSC3*, and *MAL2* were efficiently removed due to an up to 10^6^-fold depletion of leukocytes. The presence of these gene markers was observed in Parsortix™-enriched blood samples from patients with primary and recurrent gynecological cancer (32% and 14%), as well as in 86% of the metastatic breast cancer samples, at a very high specificity. In the ovarian cancer samples, *PPIC* was the most prominent gene marker, contributing to all positive cases and at least to 70% of the positive cases after pre-amplification of the respective target genes. Expanding the analytical panel up to 29 gene markers further increased the positivity rate (primary gynecological cancer: 95%, recurrent gynecological cancer: 100%, metastatic breast cancer: 92%).

The established workflow strongly improved the overall molecular analysis of the target cells by the efficient removal of contaminating cells, and, thereby offers great promise for the molecular characterization of CTCs.

## INTRODUCTION

The analysis of circulating tumor cells (CTCs) in the blood of cancer patients represents an enormous technical challenge, not only due to their low absolute numbers but also the extreme abundance of blood cells. At early stages of cancer, CTC counts were reported to be as low as 1 to 5 cells in 7.5 ml of blood [1], whereas in the metastatic setting, more than 1000 CTCs per 7.5 ml blood have been observed [2].

Prior to analysis, the standard recommendation is to increase the relative amount of CTCs. Density gradient centrifugation is one approach to enrich the blood sample for CTCs: it is easy to perform, inexpensive, suitable for large blood volumes (i.e. > 10 ml), and will not - in contrast to immune-magnetic enrichment - favor a certain CTC subtype, e.g. those cells expressing the epithelial cell adhesion molecule EpCAM. However, density gradient centrifugation will reduce the number of leukocytes only to a certain extent (by 10- to 100-fold) [3]. A further depletion of unwanted cells can be achieved by immune-magnetic capture of white blood cells using an antibody against the leukocyte-specific CD45. At first glance, this approach may be tempting, but with large blood volumes it is associated with high costs, CTCs may be trapped within the bulk of captured leukocytes, or may even bind to the magnetic beads [4].

Over the past several years, we focused on the molecular analysis of CTCs using quantitative PCR (RT-qPCR). Molecular assays for CTC detection offer many advantages, including the high sensitivity, small sample volumes, and the option to multiplex reactions and to analyze at high throughput; however, pre-analytical issues such as blood sample processing may be more crucial than with immune-fluorescent based assays [5]. In our earlier RT-qPCR-based studies we identified a 6-gene panel for the detection of CTCs in breast cancer and gynecological malignancies [6], and a further panel (comprising the cyclophilin C encoding gene *PPIC*) specifically for ovarian cancer [7]. In both studies, we used density gradient centrifugation to enrich the CTCs. The high amount of residual leukocytes in the enriched samples led to false positive results, most likely due to illegitimate transcription of the selected genes in leukocytes contaminating the enriched CTC sample. Although we were able to correlate the expression of the CTC-related molecular markers with patient outcome, we supposed that a better depletion of leukocytes would reduce the RT-qPCR background and improve both specificity and sensitivity of the molecular approach.

Further studies using molecular assays for the detection of CTC-related transcripts are primarily based on the immune-magnetic enrichment of the CTCs prior to PCR [8–12], which may achieve an appropriate depletion of leukocytes depending on the respective protocol. However, the presence of the targeted cell surface epitopes may be diminished on a subpopulation of CTCs displaying a more mesenchymal than an epithelial phenotype [13, 14]. Several studies have recently shown that the portion of epithelial-like CTCs may be low in ovarian cancer [7, 15–17], suggesting that more comprehensive enrichment strategies based on physical and not on biological CTC properties alone may be more appropriate.

Recently, ANGLE plc launched the CE-marked Parsortix™ cell separation system for the research market. The FDA clearance process for the diagnostic market in the U.S. (www.angleplc.com) is underway. The Parsortix™ system uses a micro-fluidic technology to isolate rare cells (e.g. CTCs) based on their less deformable nature and usually larger size compared to blood cells. The separation of the blood components takes place in a microscope slide sized disposable cassette, which contains a series of steps leaving a 10 µm measuring gap between the top cover and the final step. The captured cells may either be harvested for subsequent analysis, or alternatively stained within the cassette. The advantages of the technology are the antigen-independent enrichment of the target cells, the reported high purity of the captured cells, and flexibility in terms of blood volume to be processed. Thus we assumed that the Parsortix™ technology may be appropriate for the subsequent molecular analysis of the captured cells.

The aim of this study was to improve the molecular analysis of CTC-related markers by eliminating any background due to residual leukocytes. We developed a workflow which employed a density gradient pre-enrichment step, a final enrichment step using the Parsortix™ technology, and the RT-qPCR based analysis of 29 genes. The established protocol is flexible in terms of sample volume and number of gene markers analyzed, and thus offers great promise for further research on the molecular detection and characterization of CTCs in clinical studies.

## RESULTS

### Optimization of the separation workflow

**Figure 1:**
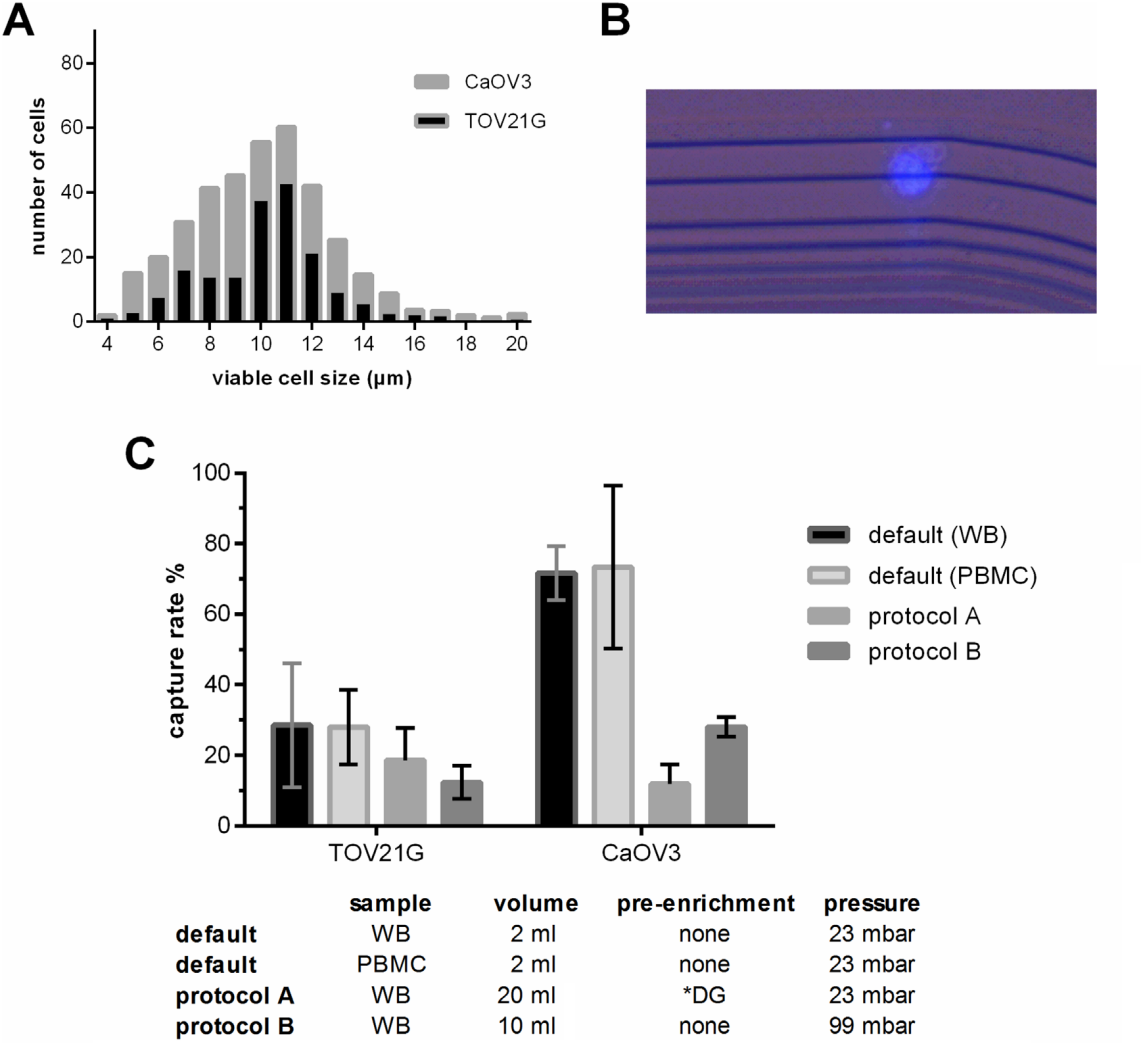
Characteristics of the applied protocols used for the enrichment of blood samples employing the Parsortix™-technology (**A**) Size distribution of viable TOV21G and CaOV3 cells. The size of the viable cells was measured using the Countess^®^ automated cell counter. (**B**) Combined fluorescence and bright field micrograph of a CellTrace Violet pre-labeled TOV21G cell captured on the separation structure in the Parsortix™ microfluidic cassette. (**C**) Capture rate of TOV21G and CaOV3 tumor cells added to the sample at equal numbers (i.e. 5 cell per ml). To test the default settings, the tumor cells were added to whole blood (WB) and to a cell fraction (PBMC) after enrichment. To test protocols A and B, the tumor cells were added to whole blood. ^*^DG density gradient centrifugation.

First we evaluated whether the Parsortix™ technology and default separation conditions (2 ml blood, 23 mbar pressure, 10 µm final step size) can be applied for ovarian cancer cells. Despite their small size, TOV21G (median diameter 11 µm, IQR 8–10 µm) and CaOV3 (median diameter 10 µm, IQR 8–12 µm) cells were captured at a mean rate of 28.4% and 71.8%, respectively (see Figure 1A–1C). The number of captured tumor cells significantly increased with the number of tumor cells added to the blood sample (TOV21G: Pearson r = 0.998, *p* = 0.045; CaOV3: Pearson r = 0.904, *p* = 0.005).

The number of captured tumor cells in a given patient sample and thus the overall sensitivity can only be increased by processing larger blood samples. To avoid long processing time, volumes larger than the default volume of 2 ml can only be processed by two ways: either by an upfront pre-enrichment step in order to remove the majority of the blood cells (protocol A), or by increasing the pressure and thus the separation flow rate (protocol B). We observed that with both protocols the overall recovery rate was lower than at the default condition; however, due to the larger sample volume, the absolute number of captured tumor cells was higher (Figure 1C). Furthermore, we observed that the overall recovery rate of TOV21G cells spiked into the blood was higher with protocol A than with B (mean 18.7% vs. 12.3 %), whereas with CaOV3 spiked samples it was exactly the opposite (11.8 % vs. 28 %).

### Evaluation of the molecular analysis using spiked samples

**Figure 2:**
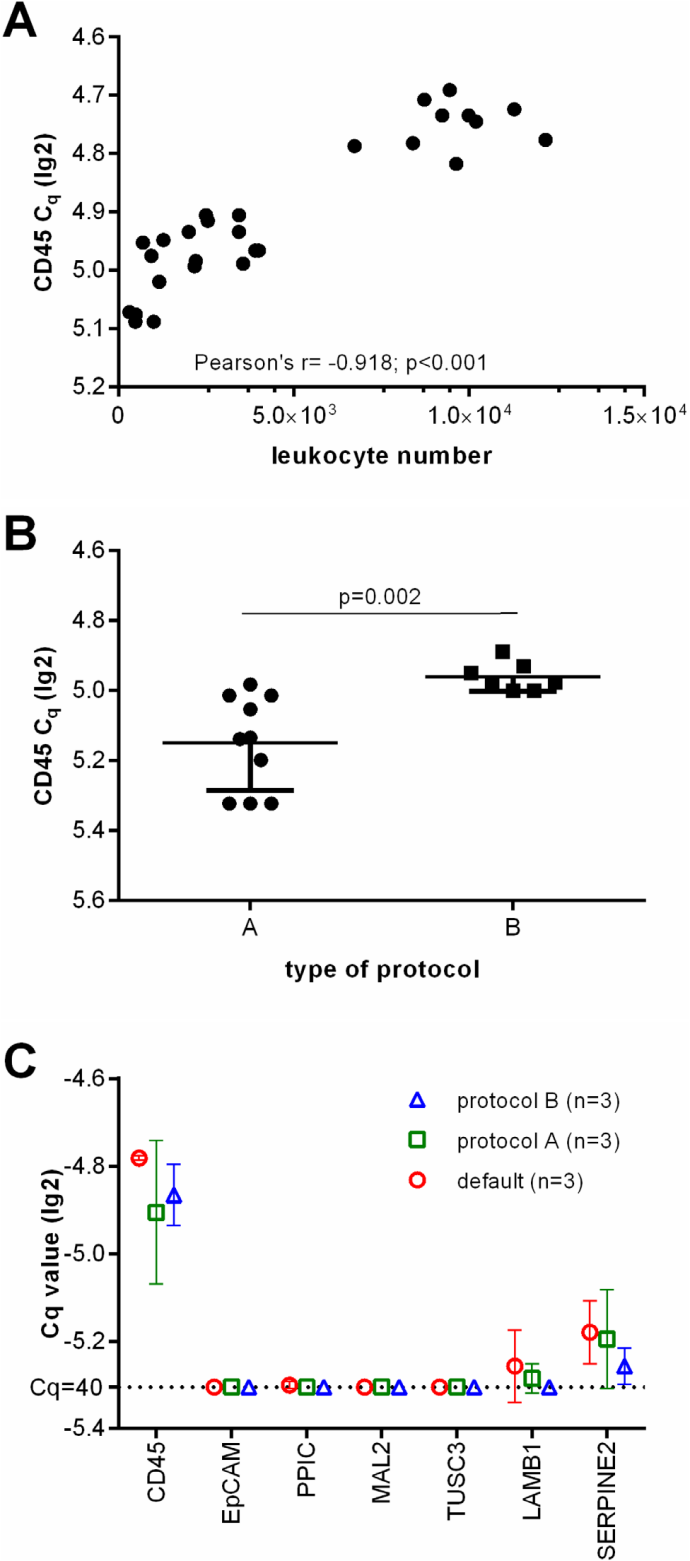
Depletion of leukocytes of the applied protocols (**A**) Showing the correlation of leukocyte numbers with lg(2) transformed Cq values of *CD45* gene expression as assessed by RT-qPCR. (**B**) lg(2) transformed Cq values of *CD45* gene expression of healthy donor blood samples processed by protocol A and B. (**C**) Relative gene marker expression levels after Parsortix™ based separation. Each three healthy donor blood samples were processed using default settings starting with 2 ml blood, protocol A starting with 20 ml blood (with an upfront density gradient enrichment), or using protocol B starting with 10 ml blood (applying an increased pressure of 99 mbar).

Next, we performed *CD45*-specific RT-qPCR in order to evaluate whether the *CD45* RNA expression levels can serve as an estimate for the leukocyte contamination. As expected, the lg2 transformed Cq values correlated with the amount of residual leukocytes in the enriched samples (Pearson r = −0.918, *p* < 0.001, see Figure 2A). Furthermore, *CD45*-specific RT-qPCR indicated that the number of residual leukocytes was significantly smaller in samples enriched by protocol A than in those which had been processed using protocol B (see Figure 2B; unpaired *t* test with Welch’s correction *p* = 0.002). Thus we assumed that protocol A would be more appropriate for the subsequent molecular analysis.

Then, we analyzed the transcript levels of the selected CTC gene markers (*MAL2*, *LAMB1*, *SERPINE2*, *PPIC*, *TUSC3*, and *EpCAM*) in each three healthy donor blood samples processed using the default settings, protocol A, and B. We did not observe an amplification signal specific for *EpCAM*, *PPIC*, *MAL2*, and *TUSC3* in any of these samples. As *LAMB1* and *SERPINE2* transcripts were detected in all of the samples (see Figure 2C), these markers were excluded from further analyses.

**Figure 3:**
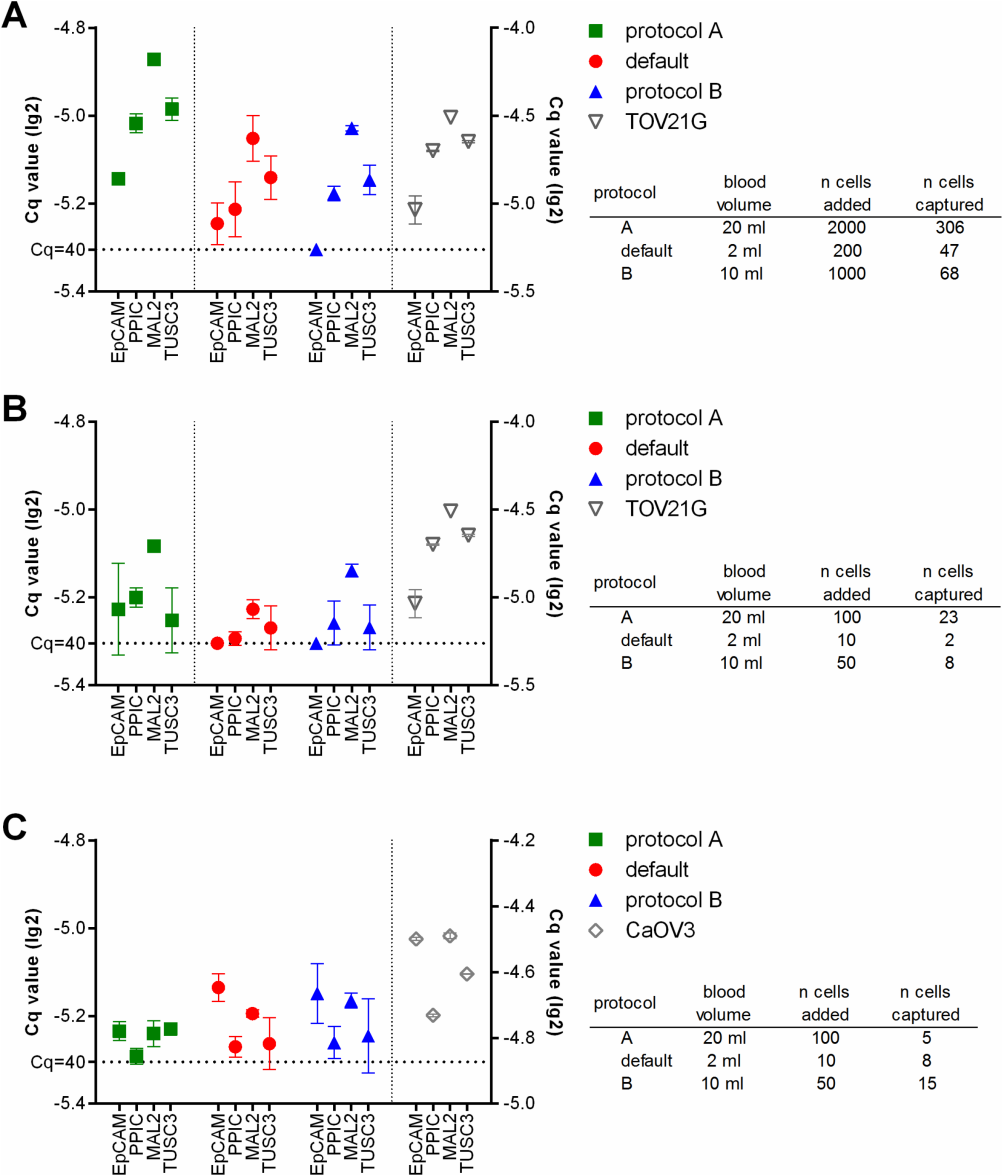
Relative gene expression levels in healthy donor blood samples spiked with TOV21G (A and B) or CaOV3 (C) tumor cells The sample volume was determined by the respective protocol used (default: 2 ml, protocol A: 20 ml, protocol B: 10 ml). The tumor cells were added to the samples at high (A: 100 cells/ml) and low (B and C: 5 cells/ml) concentrations. Then the samples were processed using the respective protocols. The RT-qPCR was done without prior pre-amplification of the targets. The resulting lg(2)-transformed Cq-values are plotted onto the left y-axis. RT-qPCR of the pure tumor cells was performed for comparison (lg(2)-transformed Cq-values plotted onto the right y-axis).

Finally we added fluorescently labeled tumor cells at defined numbers to healthy donor blood, and processed these samples using the respective protocols. After the enrichment and before lysis, the number of the tumor cells captured in the microfluidic cassette was assessed. Then RT-qPCR specific for *EpCAM*, *PPIC*, *MAL2*, and *TUSC3* was performed. The resulting Cq values correlated with the number of captured cells (Figure 3). Thus, in the samples spiked with TOV21G cells, the strongest amplification signals were observed with protocol A, in samples which contained the highest absolute number of tumor cells due to the large sample volume (Figure 3A and 3B). The amplification signal was smaller in protocol B samples and those samples which had been processed at default settings. In contrast, the strongest amplification signals in CaOV3 spiked samples were measured in samples processed using protocol B (Figure 3C). Here, the absolute number of captured cells was higher in protocol B than in protocol A (see also Figure 1C), although initially a smaller number of tumor cells had been added to the sample. Possibly, a poor recovery rate of the density gradient enrichment led to the lower number of cells in the final sample.

### Molecular analysis of patients’ blood samples, with and without pre-amplification

**Figure 4:**
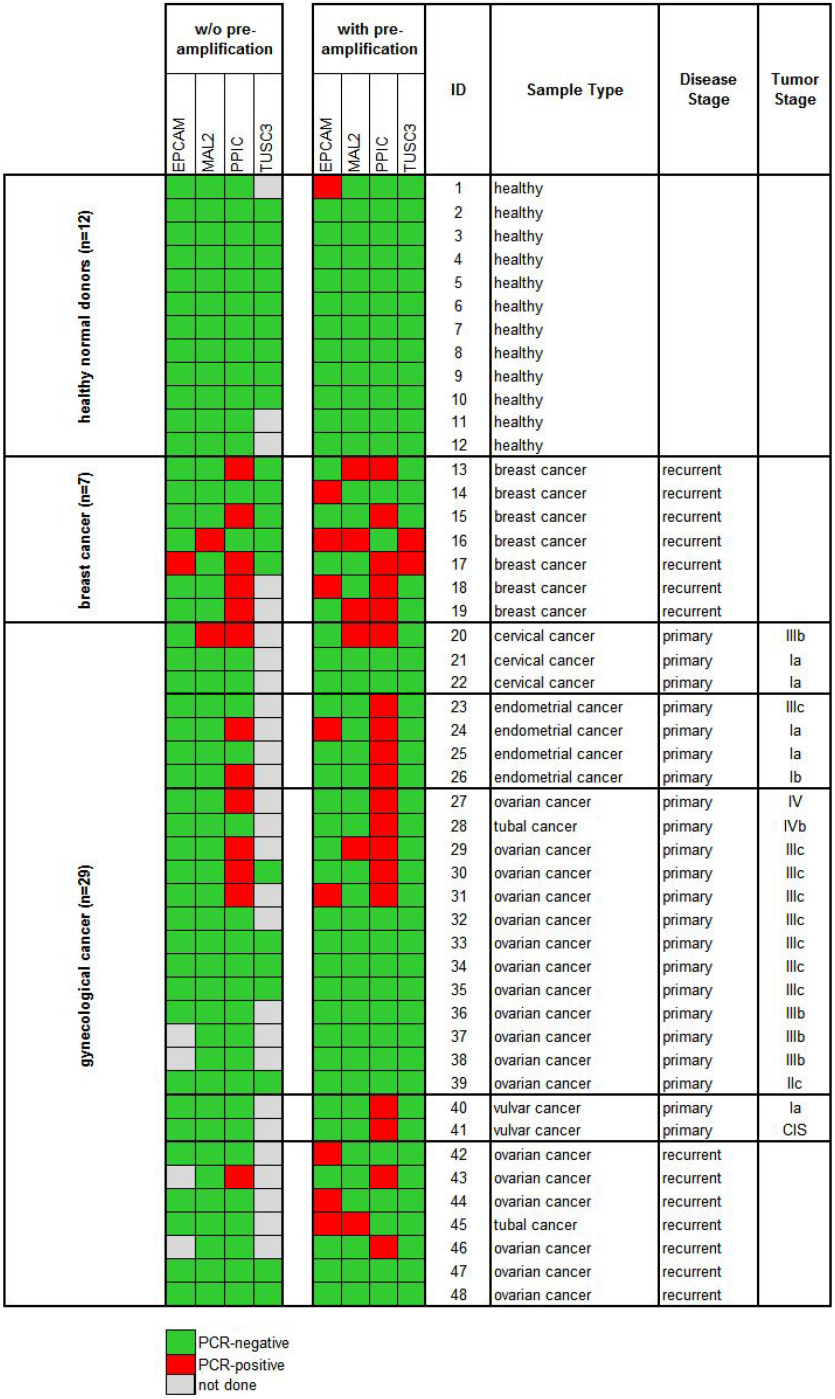
Heat map of *EpCAM*, *MAL2*, *PPIC*, and *TUSC3* gene expression analysis of Parsortix™ enriched blood samples (cohort 1) Each 20 ml blood from 12 healthy donors and 36 cancer patients were processed using protocol A (comprising an upfront density gradient enrichment). RT-qPCR was performed without (left panel) and with prior pre-amplification (right panel) of the respective transcripts. Tumor stages of the primary gynecological cancers are given according to the FIGO classification system.

In the next step, we processed blood samples from 36 cancer patients and 12 healthy donors (cohort 1) using protocol A and measured the gene expression levels of *EpCAM*, *MAL2*, *PPIC*, and *TUSC3* in the enriched cell fractions. Twenty-two samples were taken at primary diagnosis before any therapeutic intervention (cervical cancer *N* = 3, endometrial cancer *N* = 4, ovarian cancer N = 13, vulvar cancer *N* = 2), whereas fourteen samples were taken from patients with recurrent disease (ovarian cancer *N* = 7) and from patients with metastatic breast cancer (*N* = 7). The respective gene transcripts were detected in 7/22 (32%) samples taken at primary diagnosis, in 1/7 (14%) samples at recurrence, in 6/7 (86%) breast cancer samples, and in none of the 12 healthy donor samples (see Figure 4, left panel).

Then, we investigated the effects of a pre-amplification step on the measured transcript levels in the same samples. In the healthy donor samples, the transcript levels of *PPIC*, *MAL2*, and *TUSC3* were still below the detection limit of RT-qPCR. An *EpCAM* specific amplification signal was observed in one single case. Among the same 36 cancer patients included in cohort 1, after a pre-amplification step at least one of the transcripts was observed in 12/22 (55%) samples taken at primary diagnosis, in 5/7 (71%) samples at recurrence, and in all (100%) breast cancer samples (Figure 4, right panel).

### Expanding the molecular analysis by additional gene markers

**Figure 5:**
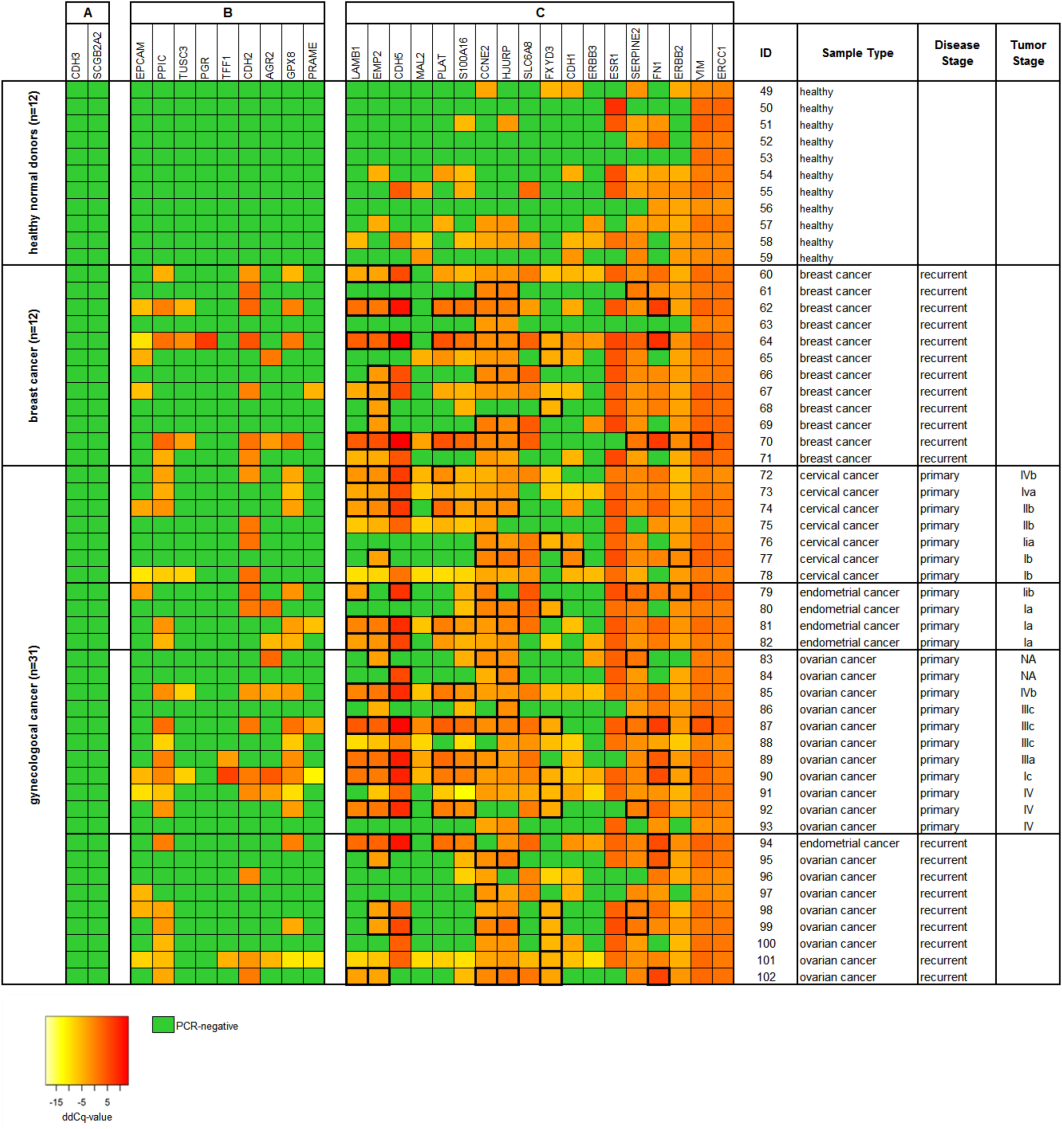
Heat map of relative gene expression levels of 29 CTC-related gene markers of Parsortix™ enriched blood samples (cohort 2) Each 20 ml blood from 11 healthy donors and 43 cancer patients were processed using protocol A (comprising an upfront density gradient enrichment). RT-qPCR was performed following a pre-amplification step of the respective transcripts. Raw Cq values were normalized to the reference gene *B2M*, resulting in dCq values. ddCq values were obtained by normalizing dCq values to a calibrator sample. The gene markers are ranked according to their respective gene expression levels in the samples from (**A**) undetected in both healthy donors and cancer patients, (**B**) undetected in healthy donors but not in cancer patients, and (**C**) detected in both healthy donors and cancer patients. ddCq levels above the two-fold standard deviation from the mean gene expression in the healthy donor samples are framed with thick black lines. Tumor stages of the primary gynecological cancers are given according to the FIGO classification system.

In a further set of 54 blood samples taken from 11 healthy female donors and from 43 cancer patients (cohort 2) we investigated the effect of additional CTC-related gene markers to the analysis (see Supplementary Table 1). In addition to the four CTC-related markers analyzed before (*PPIC*, *MAL2*, *TUSC3*, and *EpCAM*), the gene expression of further 25 markers was measured using RT-qPCR (see Figure 5). From all 29 markers analyzed, two markers (*CDH3* and *SCGB2A2)* were negative in in all samples (see Figure 5A). Nine markers (*PRAME, EpCAM, TUSC3, GPX8, PPIC, AGR2, CDH2, TFF1,* and *PGR)* were negative in all of the healthy control samples investigated. These nine markers contributed to RT-qPCR positive cases in 18/22 (82%) samples taken at primary diagnosis, in 8/12 (67%) breast cancer samples, and in 8/9 (89%) samples at recurrence (see Figure 5B).

The remaining 18 markers (see Figure 5C) were positive in the healthy control samples at various rates ranging from just 9% (*LAMB1*, 1/11) to 100% positive cases (*VIM* and *ERCC1*). However, we observed that the relative gene expression was higher in the cancer patients than in the healthy control samples. By choosing the two-fold standard deviation from the mean gene expression level in the control samples as threshold value, six from those nine cancer samples, which did not show any amplification signal related to *PGR*, *TFF1*, *CDH2*, *AGR2*, *PPIC*, GPX8, *TUSC3*, *EPCAM*, and *PRAME* were additionally assigned as being positive. Thus, the analysis of 29 markers resulted in a positivity rate of 95% in primary cancer, of 92% in breast cancer, and of 100% in recurrent cancer samples.

## DISCUSSION

In this study we established a workflow combining density gradient centrifugation and enrichment by the Parsortix™ microfluidic technology, followed by RT-qPCR analysis. The Parsortix™ technology alone or in combination with a density gradient enrichment has mainly been used for subsequent immuno-staining and single cell analysis so far [18–21]. Our workflow meets the key prerequisite for RT-qPCR based analysis of CTC-related gene markers in the obtained cell sample by efficiently removing unwanted leukocytes, which could contribute to false positives due to illegitimate transcription of the used markers. Furthermore, the protocol can be adapted to large sample volumes in order to counteract the low abundance of target cells, especially in early stage disease or in cancer types which may not primarily spread via the hematogenous route like ovarian cancer.

By combining density gradient centrifugation and the Parsortix™ separation, one third of the blood samples obtained from patients with primary ovarian cancer (cohort 1) were RT-qPCR-positive prior to pre-amplification. Of note, *PPIC* was the only gene marker which was detected in these samples. These results are in line with those obtained from our earlier study employing density gradient centrifugation alone, yielding 24% RT-qPCR-positive cases in primary ovarian cancer, mostly attributed to *PPIC*-positivity [7]. Noteworthy, in the same set of cohort 1 samples the percentage of RT-qPCR positive cases had more than doubled by adding a pre-amplification step prior to RT-qPCR. After pre-amplification, *PPIC* still contributed to 78% of the positive ovarian cancer samples in cohort 1, and to 70% in cohort 2.

Whether the observed high frequency of investigated gene markers is indeed related to the presence of CTCs or rather to other cells, like circulating endothelial cells [22] or cells of hematogenous origin still needs to be clarified in future studies [23]. However, our earlier study on the molecular characterization of CTCs in ovarian cancer had already shown the abundance and prognostic relevance of *PPIC* transcripts in density gradient enriched cell fractions [7]: *PPIC* was identified among other genes as putative CTC marker by gene expression analysis of paired tumor tissue and PBMC samples. The present results indicate that the high sample purity as provided by the Parsortix™ technology renders the possibility to expand the molecular analysis of the captured cells by employing a pre-amplification step for up to 100 different template species, and consequently to increase the chance of identifying cells displaying a deviating phenotype [24]. Thus, our study may facilitate the molecular characterization of CTCs, and help to identify further prognostic or predictive gene markers.

The amount of residual leukocytes may be similar to that of immune-magnetic enrichment employing antibodies attached to magnetic beads; however, our approach is more flexible in terms of independency from cell surface markers as enrichment targets and availability of appropriate antibodies. To the best of our knowledge, our study is the first to demonstrate the feasibility of gene expression analysis in combination with Parsortix™ for the detection of CTC-related transcripts in cancer patients. In contrast to Gorges et al. who performed single cell analysis of Parsortix™ enriched cells [20], we show that the high depletion of leukocytes provided by our approach enables the detection of CTC-related transcripts in the total amount of harvested cells, which had only been demonstrated in blood samples spiked with tumor cells but not in patient samples so far [19].

Compared with an earlier study evaluating the use of the Parsortix™ system in prostate cancer [18], the capture rate of spiked tumor cells was lower in our study using ovarian cancer cell lines. The main reason probably is the smaller size of ovarian cancer cells, which will pass the Parsortix™ separation cassette more likely than larger cells like prostate and breast cancer cells. We used two quite different cell lines for the spiking experiments, in terms of *EpCAM* protein expression, morphology, mutational status, doubling time, et cetera [25]. In addition to these biological characteristics, their respective size may at least in part contribute to the different behavior in the pre-enrichment strategies involved, which are based on the physical properties of the cells, like size, deformability, or density: Starting from whole blood, CaOV3 cells were captured more efficiently than TOV21G cells. This difference may be explained by the wider size distribution of CaOV3 cells than of TOV21G cells. In contrast, after density gradient centrifugation, the final recovery of tumor cells was worse with CaOV3 cells. Parallel spiking experiments of CaOV3 cells into whole blood before density gradient centrifugation and into a PBMC fraction obtained after centrifugation yielding similar recovery rates (see Figure 1C) indicate that the density gradient centrifugation led to the major part of CaOV3 cell loss in protocol A. However, the pattern of gene expression in the microfluidic enriched tumor cells was similar to that of the same tumor cells before enrichment (see Figure 3), suggesting that the Parsortix™ separation may not be selective for a particular cellular subtype.

We measured the gene expression levels of *CD45* as an estimate for leukocyte contamination. The observed difference in *CD45* levels between protocols A and B were in line with the total average amount of residual leukocytes, as specified by the manufacturer (i.e. 1300 vs. 3000 cells, personal communication with K. Mumford, ANGLE plc, January 2016). In concordance with another study evaluating the Parsortix™ system, the number of residual leukocytes did not depend on the sample volume, but rather on individual donors [21]. We observed that both the recovery of target cells and depletion of leukocytes were more variable in protocol A than in B. This phenomenon may be explained by varying performance either of the density gradient centrifugation or of the Parsortix™ separation at 23 mbar (protocol A), which was more prone to instability than at 99 mbar (protocol B), or of both. A weakness of our study design is that we did not compare both protocol A and B in the same patient samples; the main reason for this was the limited amount of blood, which was not sufficient for two microfluidic separations in parallel.

Based on our previous experience, our focus in the present study was on the best possible depletion of leukocytes. The significantly lower amount or leukocytes in protocol A enriched samples as compared to the ones enriched by protocol B was thus the main reason to choose protocol A for processing real patient samples. Being aware of the low yield of captured tumor cells, we are going to test further protocols for our application, in order to increase the performance of the Parsortix™ separation and the overall capture rate of the target cells. For these purposes, the density gradient centrifugation step could be replaced, e.g. by a second microfluidic separation step (as described in [21]), or separation cassettes with a smaller than 10 µm measuring critical step size could be evaluated to find the optimum balance between capture rate and sample purity. Indeed, since starting our study, the manufacturer has developed alternative cassettes with 6.5 µm and 8 µm critical step sizes, which should improve CTC capture in principle.

In conclusion, we have developed a workflow for the enrichment of CTCs, which assures the depletion of contaminating leukocytes up to 10^6^-fold and thus allows for the subsequent molecular analysis by providing a high purity of the enriched cells. Our protocol offers great promise for further research on the molecular detection and characterization of CTCs in clinical studies. Future efforts include further improving the recovery rate of the CTCs, validating the overall approach in larger sample cohorts, and evaluating the prognostic value of further CTC gene markers besides the *PPIC* gene.

## MATERIALS AND METHODS

### Blood samples

Blood samples were taken from patients suffering from gynecological malignancies, including ovarian, endometrial, and cervical cancer, and from patients with metastatic breast cancer at the General Hospital in Vienna. Control blood samples were acquired from female healthy donors without a history of cancer. All samples were taken before any therapeutic intervention after written informed consent. The blood was collected in Vacuette EDTA tubes (Greiner Bio-One) and processed within four hours. The study was approved by the Ethic Committee of the Medical University of Vienna, Austria (EK366/2003).

### Cell spiking experiments

The ovarian cancer cell lines TOV21G and CaOV3 were trypsinized at about 70% confluence and incubated with the fluorescent CellTrace Violet stain (Invitrogen) according to the manufacturer’s protocol. The mean cell diameter was measured using the Countess^®^ automated cell counter (Invitrogen). A healthy female donor blood sample was spiked with the fluorescently labeled cells by transferring single tumor cells with a pipette until a final concentration of 5 and 100 tumor cells per ml blood was achieved. The blood sample volume was 2 ml, 20 ml and 10 ml, in order to test the separation at default conditions, with protocol A and protocol B, respectively. Un-spiked blood samples from female healthy donors were used as controls. The tumor cells captured within the Parsortix™ separation cassette were counted using a fluorescence microscope to assess the capture rate (absolute number of captured tumor cells divided by total number of added tumor cells).

### Processing of blood samples

The blood samples were processed in three different ways: (i) Using the Parsortix™ default settings, 2 ml blood was processed by applying a 23 mbar pressure to push the sample through the separation cassette. For processing larger blood volumes, either protocol A or protocol B was used. (ii) With protocol A, 20 ml blood was pre-enriched by density gradient centrifugation using 15 ml Percoll (GE Healthcare; d = 1.065 g/ml, 305 mOsm/kg) in Sepmate^®^ tubes (Stemcell Technologies). After centrifugation at 4°C at 1350x g for 20 mins with disabled brake, the enriched cells in the top layer were washed with PBS and further processed using the Parsortix™ technology by applying a 23 mbar pressure. (iii) With protocol B, the 10 ml whole blood sample (diluted with 10 ml PBS) was directly processed without any further pre-enrichment at a 99 mbar pressure. With each of the three protocols, Parsortix™ separation cassettes with a 10 µm gap size were used. After separation the cassette was either visually examined under a microscope to count fluorescently labeled tumor cells (spiking experiments), and/or was flushed with 350 µl RLT lysis buffer (Qiagen) to retrieve the enriched cells for subsequent molecular analysis. The cell lysates were stored at −20°C until RNA extraction.

### Selection of target genes

The RT-qPCR markers were selected based on our earlier studies which compared the gene expression of healthy control blood samples and tumor cell lines [6], and of paired tumor tissue and PBMC samples using microarrays [7]. Both studies aimed at identifiying transcripts which may indicate the presence of CTCs in patient blood samples. In that former studies we validated 384 differentially expressed genes with RT-qPCR. Here, we selected just a few of the genes most abundantly expressed in the ovarian cancer cell lines CaOV3 and TOV21G, which were used for the spiking experiments. In addition to these gene markers (*MAL2*, *LAMB1*, *SERPINE2*, *PPIC*, and *TUSC3*), we selected *EpCAM* as known marker specific for epithelial cells, *CD45* as a marker for residual leukocyte content, and ß-2-microglobulin (*B2M*) as reference gene. In those experiments with an upfront pre-amplification step (which allows the analysis of up to 100 transcripts) we included further gene markers which had been investigated in our earlier studies and which had been previously described as being specific for epithelial or mesenchymal cells (Supplementary Table 1).

### Molecular analysis

Total RNA was extracted from the cell lysates using the RNeasy Micro Kit (Qiagen) without DNase treatment. The total RNA amount was incubated with random nonamers (Sigma-Aldrich) at 70°C for 5 mins. After cooling to 4°C, M-MLV Reverse Transcriptase, RNase H Minus, Point Mutant (Promega), reaction buffer, and RNAsin (Promega) were added. The 20 µl reaction mix was incubated at 25°C for 15 mins, then at 45°C 50 mins and at 55°C 10 mins. Pre-amplification was performed using the same TaqMan^®^ assays as with RT-qPCR and the TaqMan^®^ PreAmp Mastermix according to the manual (Life Technologies). The uniformity of the pre-amplification of the target genes relative to *CDKN1B* as reference gene was assessed according to the manual. RT-qPCR was done in duplicates in a 10 µl total reaction volume on the ViiA7 Real-Time PCR System using exon spanning TaqMan^®^ assays and TaqMan^®^ Universal Mastermix II (Life Technologies) with default thermal cycling parameters (50°C 2 mins; 95°C 10 mins; 40 cycles at 95°C 15 s, 60°C 1 min). Raw data were analyzed using the ViiA7 Software v1.1 with automatic threshold setting and baseline correction. dCq value were calculated by normalizing the mean Cq value of gene X to the mean Cq of *B2M*. ddCq values were calculated by normalizing dCqs to a reference sample. If an amplification signal was observed in the healthy donor samples, a cut-off threshold value was calculated by adding the twofold standard deviation of the mean observed ddCq value in the control samples [26]. A patient sample was then assigned positive if the ddCq value of the respective gene marker was beyond that threshold.

### Statistics

Graphs and statistics were done using GraphPad Prism version 6.01.

## SUPPLEMENTARY MATERIALS TABLE



## Abbreviations

CTC: circulating tumor cell
Cq: quantitation cycle
CD45: cluster of differentiation 45
EpCAM: epithelial cell adhesion molecule
LAMB1: laminin; beta 1
MAL2: mal T-cell differentiation protein 2 (gene/pseudogene)
PBMC: peripheral blood mononuclear cells
PPIC: peptidylprolyl isomerase C (cyclophilin C)
RT-qPCR: reverse transcription quantitative polymerase chain reaction
SERPINE2: Serpin family E member 2
TUSC3: tumor suppressor candidate 3
