# Supplementary material for: Efficient leukocyte depletion by a novel microfluidic platform enables the molecular detection and characterization of circulating tumor cells
Source: Oncotarget. 2017 Nov 28;9(1):812–23. doi: 10.18632/oncotarget.22549 (PMC5787513; doi:10.18632/oncotarget.22549)
Supplement: Supplementary file 1 [file oncotarget-09-812-s001.pdf]

Efficient leukocyte depletion by a novel microfluidic platform enables the molecular detection and characterization of circulating tumor cells

SUPPLEMENTARY MATERIALS

Supplementary Table 1: CTC-related gene markers

Symbol	Gene Name	TaqMan® Assay ID
AGR2	anterior gradient 2	Hs00180702_m1
CCNE2	cyclin E2	Hs00372959_m1
CDH1	cadherin 1; type 1	Hs01023894_m1
CDH2	cadherin 2; type 1	Hs00169953_m1
CDH3	cadherin 3; type 1	Hs00354998_m1
CDH5	cadherin 5; type 2	Hs00901465_m1
EMP2	epithelial membrane protein 2	Hs00171315_m1
EPCAM	epithelial cell adhesion molecule	Hs00158980_m1
ERBB2	v-erb-b2 avian erythroblastic leukemia viral oncogene homolog 2	Hs00170433_m1
ERBB3	v-erb-b2 avian erythroblastic leukemia viral oncogene homolog 3	Hs00176538_m1
ERCC1	excision repair cross-complementing rodent repair deficiency; complementation group 1 (includes overlapping antisense sequence)	Hs01012158_m1
ESR1	estrogen receptor 1	Hs00174860_m1
FN1	fibronectin 1	Hs00365058_m1
FXYD3	FXYD domain containing ion transport regulator 3	Hs00254211_m1
GPX8	glutathione peroxidase 8 (putative)	Hs00380670_m1
HJURP	Holliday junction recognition protein	Hs00251144_m1
LAMB1	laminin; beta 1	Hs00158620_m1
MAL2	mal; T-cell differentiation protein 2 (gene/pseudogene)	Hs00294541_m1
PGR	progesterone receptor	Hs00172183_m1
PLAT	plasminogen activator; tissue	Hs00263492_m1
PPIC	peptidylprolyl isomerase C (cyclophilin C)	Hs00181460_m1
PRAME	preferentially expressed antigen in melanoma	Hs00196132_m1
S100A16	S100 calcium binding protein A16	Hs00293488_m1
SCGB2A2	secretoglobin; family 2A; member 2	Hs00267190_m1
SERPINE2	serpin peptidase inhibitor; clade E (nexin; plasminogen activator inhibitor type 1); member 2	Hs00299953_m1
SLC6A8	solute carrier family 6 (neurotransmitter transporter); member 8	Hs00373917_g1
TFF1	trefoil factor 1	Hs00170216_m1
TUSC3	tumor suppressor candidate 3	Hs00954406_m1
VIM	vimentin	Hs00185584_m1
